# Presence of blood in gastric juice: A sensitive marker for gastric cancer screening in a poor resource setting

**DOI:** 10.1371/journal.pone.0205185

**Published:** 2018-10-15

**Authors:** Violet Kayamba, Kanekwa Zyambo, Paul Kelly

**Affiliations:** 1 Tropical Gastroenterology & Nutrition group, Department of Internal Medicine, University of Zambia School of Medicine, Lusaka, Zambia; 2 Blizard Institute, Barts & The London School of Medicine and Dentistry, Queen Mary University of London, London, United Kingdom; University Hospital Llandough, UNITED KINGDOM

## Abstract

**Background:**

Gastric cancer survival rates in Africa are low as many cases are diagnosed late. Currently, there are no inexpensive, non-invasive and simple techniques that can be employed in poor resource settings for early case detection. In this study, we explored the possibility using blood in gastric juice as a screening tool to identify patients requiring referral for endoscopy.

**Methods:**

The study was conducted at the University Teaching Hospital endoscopy unit in Lusaka, Zambia. During esophagogastroduodenoscopy, gastric juice was aspirated and the pH determined using pH paper test strips. The presence of blood was tested using urinalysis reagent strips.

**Results:**

We enrolled 276 patients; 147(53%) were female and median age was 49 years (IQR 40–64 years). The presence of blood was associated with mucosal lesions, [OR 2.1; 95% CI 1.2–3.7, *P* = 0.004]. It was also associated with gastric cancer, [OR 6.7; 95% CI 2–35, *P* = 0.0005], even at 1:10 and 1:100 dilutions, [OR 5.4; 95% CI 2.3–13.8, *P*<0.0001] and [OR 9.1; 95% CI 3.5–23, *P*<0.0001] respectively. The sensitivity for gastric cancer detection using blood in gastric juice was 91% and the specificity was 41%. Analysis using the intensity of blood in gastric juice yielded an area under the receiver operating characteristic curve of 0.78; 95% CI 0.71–0.86 with a sensitivity of 79% and a specificity of 77%.

**Conclusions:**

The presence of blood in gastric juice is associated with gastric mucosal lesions. It has a high sensitivity but low specificity for gastric cancer detection.

## Background

Gastric cancer is the third leading cause of cancer deaths worldwide with more than 70% of the cases occurring in developing countries [1,2]. On average, the five-year survival for gastric cancer is 25%, but rates less than 5% have been reported in some African countries [3, 4]. The stage of disease at initial diagnosis is a major determinant of outcome, and in Africa gastric cancer is often diagnosed late [5, 6].

Early gastric cancer diagnosis is a challenge in low-resource settings as endoscopy is expensive, invasive and requires trained personnel. In addition, ordinary white light endoscopy with histology, which is the gold standard for gastric cancer, has low sensitivity for detection of early gastric lesions [7]. There are several innovative strategies being developed to enhance the sensitivity of endoscopic biopsies such as confocal endomicroscopy, narrow band imaging, magnifying endoscopy with blue laser among others [8, 9, 10, 11], but application of these techniques is not possible in many parts of Africa. Less invasive strategies being evaluated make use of easily obtained samples such as blood, urine and saliva. Some of these include circulating tumour cells [12], cytokines [13], and tumour markers [14]. More recently, there have been reports of promising gastric cancer biomarkers detected in gastric juice, particularly long non-coding RNA [15, 16], micro RNA [17] and tryptophan metabolites [18]. However, many of these strategies employ molecular and highly technical approaches that are not currently feasible in poor resource settings with scanty sources of electricity and clean water. Therefore, cheap, less invasive and technically simpler methods are urgently needed for early gastric cancer detection in Africa.

Another hindrance to early gastric cancer detection is the lack of discriminatory clinical features. Alarm symptoms including weight loss, haematemesis, melaena, dysphagia, and anaemia are usually not apparent in early disease [19]. Others such as abdominal pain and persistent dyspepsia [19] are highly non-specific. This possesses diagnostic challenges for health care providers in centers that do not have endoscopic facilities. There is a need for a simple technique, preferably with a high negative predictive value that would enable clinicians in these low-resource rural settings to determine which individuals need to travel to more specialized centers for endoscopy.

We therefore set out to test the possibility of employing readily available diagnostic tools to predict which patients are likely to have gastric cancer. In this paper, we set out to test a concept that could subsequently be applied by unspecialized health care workers in very remote and low resourced settings. We hypothesized that testing gastric juice for the presence of blood would reveal which individuals have gastric mucosal lesions and therefore in need for endoscopic evaluation. The University of Zambia Biomedical Research Ethics committee, reference number 000-03-16, approved this study.

## Methods

### Patient recruitment

The study was conducted between July 2016 and November 2017 at the University Teaching Hospital (UTH) endoscopy unit in Lusaka, Zambia. All consenting adults above the age of 18 years were considered for recruitment. All participants gave written and well informed consent. Excluded were those with large bleeding oesophageal varices, occluding oesophageal lesions and previously confirmed or treated for gastric or oesophageal cancer. These patients all fasted overnight prior to the procedures. During the oesophagogastroduodenoscopy (OGD), contents of the biopsy channel were cleared and gastric juice aspirated using a 10 ml syringe. Aspiration of gastric juice was done immediately upon entering the stomach. The rest of the upper gastrointestinal tract was then examined following the standard of care. Biopsies were taken from mucosal lesions seen. After the OGD, gastric juice pH was measured using pH paper test strips (Sigma Chemical Company St Louis, USA). An aliquot of the juice was saved at -80°C for further experiments.

### Description of endoscopic findings

Mucosal lesions diagnosed during the OGD included the following;

1. Gastric cancer, with fungating lesion seen
2. Gastric, duodenal or oesophageal ulcers, with a clean base and edges
3. Mucosal inflammation without ulceration
4. Oesophageal varices

### Determination of blood in gastric juice using urinalysis reagent strips

To test for the presence of blood in gastric juice, we used urinalysis reagent strips (ACON laboratories San Diego, USA). These strips test for the qualitative and semi-quantitative of analytes such as blood in urine with the ability to detect free haemoglobin as low as 0.018–0.06 mg/dL or 5–10 erythrocytes per μL. The test for blood is based on peroxidase-like activity of haemoglobin resulting in colour changes ranging from orange to green to dark blue, which is read manually. Depending on the colour change, the presence of blood was recorded on an ordinal scale as 1, 2 or 3 plus (+). Samples with 2+ or 3+ were considered to have a high intensity of blood. As gastric juice has much lower pH than urine, we conducted preliminary experiments to determine the influence of pH on detection of blood using these strips (S1 Table). We used Hydrochloric acid solutions and a pH of 1 or 2 showed colour change to blue without the presence of blood. In subsequent experiments, we therefore diluted samples with pH less than 3 in order to reduce the acidity, as follows:

1. Gastric juice with pH 2.5; 1:10 dilution resulting in pH 3.5, (n = 9)
2. Gastric juice with pH 2; 1:10 dilution resulting in pH 3, (n = 20)
3. Gastric juice with pH 1.5: 1:100 dilution resulting in pH 3.5, (n = 29)
4. Gastric juice with pH 1: 1:100 dilution resulting in pH 3, (n = 7)

Data were analysed by both including and then excluding these diluted samples.

All samples were then re-analysed at 1:10 and 1:100 dilutions.

### Data analysis

We used proportions, medians and interquartile ranges to summarise categorical and continuous variables respectively. Binary variables were compared using Fisher’s exact test and presented as odds ratios with 95% confidence intervals. The Kruskal-Wallis test was used to compare continuous variables. In all instances, a two-sided *P* value of <0.05 was considered statistically significant. Statistical analysis was done in STATA 13 (College Station, TX, USA).

## Results

### Basic patient characteristics

**Fig 1:**
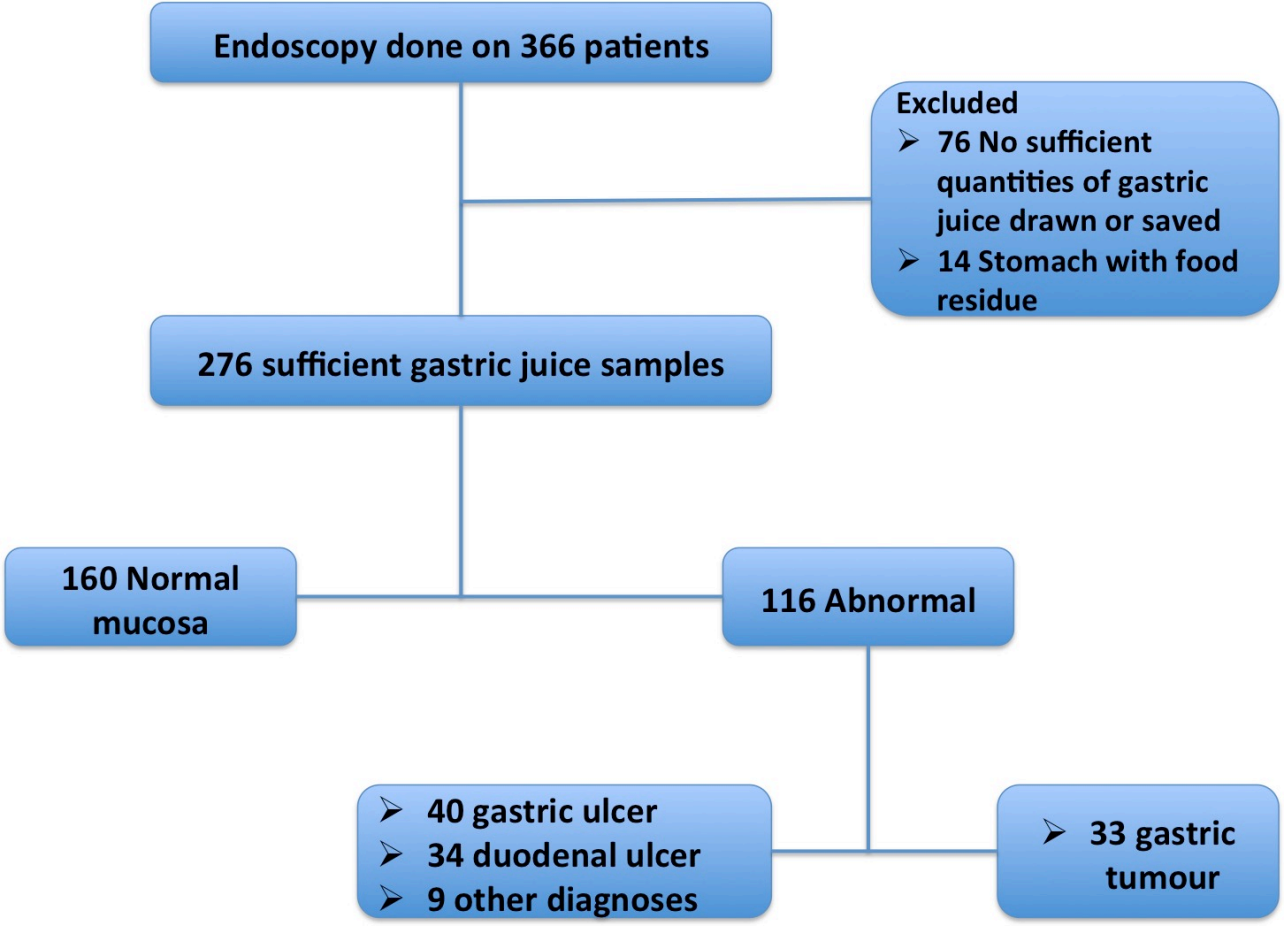
A flow chart showing patient recruitment and sample collection.

Patient inclusion was as outlined in Fig 1.

A total of 276 patients, of whom 147(53%) were female and the median age was 49 years (IQR 40–64 years) were enrolled. Of these patients, 116(42%) had mucosal abnormalities with 40(34%) benign gastric ulcers, 34(29%) duodenal ulcers and 33(28%) gastric tumours. The remaining 9(8%) had oesophageal abnormalities, polyps or non-specific inflammation. Of the 33 patients with gastric tumours, 27(82%) had adenocarcinoma. Patients with mucosal abnormalities were significantly older and more likely to present with blood loss or anaemia, (Table 1). In addition, patients with gastric tumours were more likely to be unemployed and have less than secondary education.

**Table 1:** Comparison of the basic characteristics of patients with normal and abnormal oesophagogastroduodenoscopy findings.

	Abnormal OGD n = 116: n(%)	Normal OGD n = 160: n(%)	OR; 95% CI	*P*
Female	59(51)	88(55)	0.8; 0.5–1.4	0.542
Age in years, n (IQR)	57(45–69)	45(39–55)	-	<0.001
Residence in capital city	70(60)	116(73)	0.6; 0.3–0.95	0.026
No employment	41(36)	38(24)	1.7; 1–3.1	0.043
No secondary education	55(47)	44(28)	2.3; 1.4–4.1	0.001
History of blood loss or anemia	34(29)	20(13)	2.9; 1.5–5.7	0.001
History of abdominal pain	75(65)	122(76)	0.6; 0.3–1	0.043
History of vomiting	12(10)	12(8)	1.4; 0.6–3.6	0.517
History of acid suppressing drugs	71(65)	108(75)	0.6; 0.3–1	0.07
Current smoker	6(6)	10(10)	0.8; 0.2–2.5	0.80
Current intake of alcohol	30(27)	32(21)	1.4; 0.7–2.6	0.30
	Gastric tumour n = 33: n (%)	No gastric tumour n = 243: n (%)	OR; 95% CI	P
Female	18(55)	129(53)	1.1; 0.5–2.4	1.00
Age in years, n (IQR)	63(53–71)	48(39–60)	-	<0.001
Residence in capital city	16(48)	170(70)	0.4; 0.2–0.9	0.016
No employment	16(48)	63(26)	2.6; 1.2–5.9	0.013
No secondary education	18(55)	81(33)	2.4; 1.1–5.4	0.021
History of blood loss or anemia	9(27)	45(19)	1.7; 0.6–4	0.245
History of abdominal pain	20(61)	177(73)	0.6; 0.3–1.3	0.154
History of vomiting	5(15)	19(9)	2.1; 0.6–6.4	0.183

### Blood in gastric juice as a marker of gastric pathology

Overall, 95/276(34%) of the patients had hypochlorhydria with pH greater than 4. 179/276(65%) had history of having taken acid suppressing medication within two weeks of enrolment. The median pH for the patients with normal OGD was 6 while it was 5.5 in those with abnormalities, *P* = 0.15. 57/276(21%) of the patients had pH less than 3 including 7 with pH 1, 21 pH 1.5, 20 pH 2 and 9 pH 2.5. All these were diluted as outlined in the methods above. Excluding these samples from the analysis did not alter the results (data not shown).

The presence of blood in gastric juice was significantly associated with abnormal endoscopic findings, even at 1:10 and 1:100 dilutions, (Table 2).

**Table 2:** The presence of blood in gastric juice is associated with abnormal oesophagogastroduodenoscopy.

Gastric juice	Abnormal OGD n = 116: n(%)	Normal OGD n = 160: n(%)	OR; 95% CI	P
Undiluted	85(73)	90(56)	2.1(1.2–3.7)	0.004
1:10 dilution	61(52)	45(28)	2.7(1.6–4.7)	<0.001
1:100 dilution	22(19)	10(6)	3.4(1.5–8.5)	0.001
Gastric juice	Gastric tumour n = 33: n (%)	No gastric tumour n = 243: n (%)	OR; 95% CI	P
Undiluted	30(91)	145(60)	6.7; 2–35.3	0.0005
1:10 dilution	24(72)	79(33)	5.4; 2.3–13.8	<0.0001
1:100 dilution	13(39)	16(7)	9.1; 3.5–23.3	<0.0001

**Fig 2:**
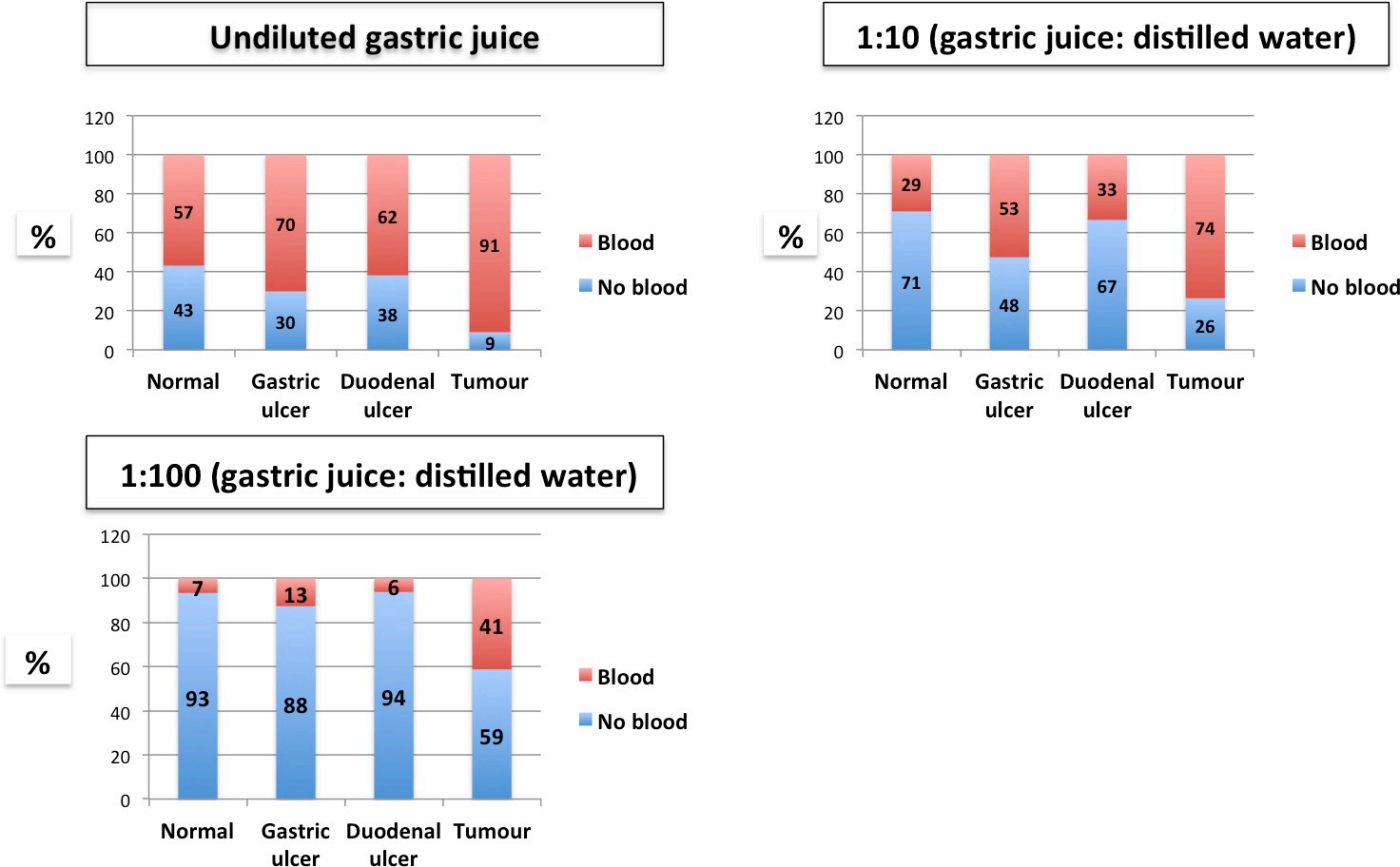
Presence of blood in gastric juice, stratified by *oesophagogastroduodenoscopy* diagnosis.

However, further dilution of the gastric juice samples did not significantly improve the test output as it reduced the sensitivity, (Fig 2).

Having a history of blood loss or anaemia was not associated with presence of blood in gastric juice, [OR 0.9; 95% confidence interval (CI) 0.5–1.7, *P* = 0.75].

### Blood in gastric juice as a marker of gastric cancer

The association between gastric cancer and blood in gastric juice was statistically significant both for neat and diluted samples, (Table 2). A high intensity of blood in gastric juice defined by colour change signifying 2+ or 3+ was higher in patients with gastric cancer 26(79%) than in those without 55(23%), [OR 12.7; 95% CI 5–36, *P*<0.0001]. The sensitivity for cancer detection using blood in neat gastric juice was 91% with a specificity of 41%. The area under the receiver operating characteristic (ROC) curve for gastric cancer detection was 0.66 with a 95% CI of 0.6–0.72. Considering the intensity of blood (as defined above) in gastric juice for detection of gastric cancer, the area under the ROC curve was 0.78 with a 95% CI of 0.71–0.86. The sensitivity of this approach was 79% with a specificity of 77%.

## Discussion

In this study, we explored the feasibility of a simple method for identifying individuals likely to have gastric mucosal lesions and therefore in need of endoscopic evaluation. Our results show that testing for blood in gastric juice is sensitive for gastric cancer detection but the specificity is low. This strategy could assist health care providers in low-resource rural settings. To test the concept, we collected the gastric juice using endoscopy but it could also be obtained using a thin nasogastric tube as a simple bedside sample collection tool. Alternatively, the patient could swallow a tethered capsule for detection of haemoglobin. This is a low cost novel idea that can be used by unskilled health workers in rural settings. These health workers are very frequently faced with patients presenting with dyspepsia but without alarm symptoms suggestive of cancer. Many such patients do not have gastric mucosal lesions but some could have early gastric lesions. In such situations, testing for blood in gastric juice could help healthcare providers prioritise patients in greatest need of endoscopy.

Gastric cancer carries a poor prognosis with one-year mortality of more 80% reported from Zambia [4]. One of the major contributors to poor outcomes is late diagnosis and there is a paucity of diagnostic facilities in sub-Saharan Africa. There are no specific symptoms for early gastric cancer and in many cases affected patients are asymptomatic. Discernable gastric cancer symptoms such as weight loss, anaemia and haematemesis only become obvious with advanced disease. This compounds the diagnostic challenge faced by health care workers in rural settings without access to endoscopic services. Endoscopy with biopsy is the gold standard for gastric cancer diagnosis, but it is expensive, invasive and requires trained personnel making difficult to implement on a population level in most sub-Saharan countries. In Korea, a high gastric cancer incidence country, its national screening programme using endoscopy was shown to significantly reduce the likelihood of dying from gastric cancer [20]. Such a programme however cannot be implemented in regions with widely scattered endoscopy facilities. A more effective strategy therefore, would be to direct the scarce resources to individuals most likely to have early gastric lesions. In Zambia for example, a 38-year audit showed that close to 70% of the endoscopies done were non-revealing [21]. With the correct screening tool, it could have been possible to identify patients who were more likely to have pathology and in need of endoscopy. We do acknowledge that this might not be an acceptable strategy in better-resourced centres, but in those struggling to maintain expensive endoscopic equipment, there is merit in finding ways of reducing unnecessary demand for endoscopy. We are in no way suggesting that this strategy could replace endoscopy but has the potential of being applied as an initial screening tool for individuals without alarm symptoms.

Recently, there has been a surge of publications on non-invasive ways of diagnosing gastric cancer and its premalignant lesions using easily obtained specimens such as urine or blood but these use molecular technologies, which are difficult to set up in rural Africa. A simpler bedside test, which can deliver the results instantly, would be more useful. The use of urinary reagent strips is one such strategy as they are fairly cheap and readily available even in the most basic centres, using health workers with very basic training. Zambia, for example has less than half of the World Health Organisation’s recommended Human Resource for Health workforce [22], a situation not dissimilar from other sub-Saharan African countries. There is need to therefore conduct more research on simple approaches to difficult health problems.

Colak et al. suggested the use of faecal occult blood-transferrin test on gastric aspirate to diagnose upper gastrointestinal bleeding but their study was done on patients who had already reported a history of haematemesis [23]. There was a recent publication reporting the potential use of a capsule system for detecting gastric occult blood but it relied on computerized data interpretation, which similarly demands considerable resources [24].

The limitation of this study is that endoscopic intubation can sometimes cause some mucosal abrasions. The urinary reagent strips used in this study were sensitive even to small amounts of blood. This could have lead to over estimation of patients with blood in gastric juice. The weakness of this approach is low specificity. Our data however, suggest a reduction of normal endoscopic findings by 40%.

There was a high proportion of hypochlorhydria in our patient cohort and use of acid suppressing medication could have influenced this observation. However, a previous community based study in Zambia also showed a similarly high proportion of hypochlorhydria of 37% [25]. Our preliminary experiments showed that the urinary reagent strips would not be applicable at low pH levels. Patients could be put on gastric acid suppressants such as proton pump inhibitors or oral acid buffers could be taken before the test.

## Conclusion

The presence of blood in gastric juice is associated with gastric cancer and other mucosal lesions. The sensitivity of the approach for gastric cancer detection is high but with a low specificity.

## Supporting information

S1 Table Testing the utility of urinary reagent strips for detection for blood in samples of with different pH levels. (DOCX)

## References

[1] Bray F, Ren JS, Masuyer E, Ferlay J. Estimates of global cancer prevalence for 27 sites in the adult population in 2008. Int J Cancer. 2013 3 1;132(5):1133–45. 10.1002/ijc.27711 22752881

[2] Fitzmaurice C, Dicker D, Pain A, Hamavid H, Moradi-Lakeh M, et al The Global Burden of Cancer 2013. Global Burden of Disease Cancer Collaboration, JAMA Oncol. 2015 7;1(4):505–27. 10.1001/jamaoncol.2015.0735 26181261 PMC4500822

[3] Sankaranarayanan R, Swaminathan R, Lucas E. Cancer survival in Africa, Asia, the Caribbean and Central America (SurvCan). IARC Scientific Publications volume 162, ISBN 978-92-832-2162-3, Lyon, International Agency for Research on Cancer, 2011. 21675403

[4] Asombang AW, Kayamba V, Turner-Moss E, Banda L, Trinkaus K, Colditz G et al Gastric malignancy survival in Zambia, Southern Africa: A two year follow up study, *Medical Journal of Zambia*, 2014 Vol. 41, No 1. PMC10511215 37731812

[5] Benamro F, Sartorius B, Clarke DL, Anderson F, Loots E, Olinger L. The spectrum of gastric cancer as seen in a large quaternary hospital in KwaZulu-Natal, South Africa. S Afr Med J. 2017 1 30;107(2):130–133. 10.7196/SAMJ.2017.v107i2.11383 28220739

[6] Ahmed A, Ukwenya AY, Makama JG, Mohammad I. Management and outcome of gastric carcinoma in Zaria, Nigeria. Afr Health Sci. 2011 9;11(3):353–61. 22275924 PMC3261017

[7] Zhang Q, Wang F, Chen ZY, Wang Z, Zhi FC, Liu SD et al Comparison of the diagnostic efficacy of white light endoscopy and magnifying endoscopy with narrow band imaging for early gastric cancer: a meta-analysis.Gastric Cancer. 2016 4;19(2):543–52. 10.1007/s10120-015-0500-5 25920526

[8] Yoshimizu S, Yamamoto Y, Horiuchi Y, Omae M, Yoshio T, Ishiyama A et al Diagnostic performance of routine esophagogastroduodenoscopy using magnifying endoscope with narrow-band imaging for gastric cancer. Dig Endosc. 2018 1;30(1):71–78. 10.1111/den.12916 28685858

[9] Kimura-Tsuchiya R, Dohi O, Fujita Y, Yagi N, Majima A, Horii Y et al Magnifying Endoscopy with Blue Laser Imaging Improves the Microstructure Visualization in Early Gastric Cancer: Comparison of Magnifying Endoscopy with Narrow-Band Imaging. Gastroenterol Res Pract. 2017;2017:8303046 10.1155/2017/8303046 28947900 PMC5602650

[10] Kayamba V, Shibemba A, Zyambo K, Heimburger DC, Morgan DR, Kelly P. High prevalence of gastric intestinal metaplasia detected by confocal laser endomicroscopy in Zambian adults. PLoS One. 2017 9 8;12(9):e0184272 10.1371/journal.pone.0184272 28886101 PMC5590914

[11] Zuo XL, Li Z, Li CQ, Zheng YY, Xu LD, Chen J et al Probe-based endomicroscopy for in vivo detection of gastric intestinal metaplasia and neoplasia: a multicenter randomized controlled trial. Endoscopy. 2017 11;49(11):1033–1042. 10.1055/s-0043-115382 28753702

[12] Kang HM, Kim GH, Jeon HK, Kim DH, Jeon TY, Park DY et al Circulating tumor cells detected by lab-on-a-disc: Role in early diagnosis of gastric cancer. PLoS One. 2017 6 29;12(6):e0180251 10.1371/journal.pone.0180251 28662130 PMC5491173

[13] Sánchez-Zauco N, Torres J, Gómez A, Camorlinga-Ponce M, Muñoz-Pérez L, Herrera-Goepfert R, Medrano-Guzmán R et al Circulating blood levels of IL-6, IFN-γ, and IL-10 as potential diagnostic biomarkers in gastric cancer: a controlled study. BMC Cancer. 2017 5 30;17(1):384 10.1186/s12885-017-3310-9 28558708 PMC5450104

[14] Chen C, Chen Q2, Zhao Q2, Liu M2, Guo J2. Value of Combined Detection of Serum CEA, CA72-4, CA19-9, CA15-3 and CA12-5 in the Diagnosis of Gastric Cancer. Ann Clin Lab Sci. 2017 5;47(3):260–263. 28667025

[15] Yang Y, Shao Y, Zhu M, Li Q, Yang F, Lu X et al Using gastric juice lncRNA-ABHD11-AS1 as a novel type of biomarker in the screening of gastric cancer. Tumour Biol. 2016 1;37(1):1183–8. 10.1007/s13277-015-3903-3 26280398

[16] Shao Y, Ye M, Jiang X, Sun W, Ding X, Liu Z et al Gastric juice long noncoding RNA used as a tumor marker for screening gastric cancer. Cancer. 2014 11 1;120(21):3320–8. 10.1002/cncr.28882 24986041

[17] Yu X, Luo L, Wu Y, Yu X, Liu Y, Yu X et al Gastric juice miR-129 as a potential biomarker for screening gastric cancer. Med Oncol. 2013 3;30(1):365 10.1007/s12032-012-0365-y 23307240

[18] Choi JM, Park WS, Song KY, Lee HJ, Jung BH. Development of simultaneous analysis of tryptophan metabolites in serum and gastric juice—an investigation towards establishing a biomarker test for gastric cancer diagnosis. Biomed Chromatogr. 2016 12;30(12):1963–1974. 10.1002/bmc.3773 27240299

[19] Cancer Research UK, http://www.cancerresearchuk.org/about-cancer/stomach-cancer/symptoms, accessed on 20th November 2017.

[20] Jun JK, Choi KS, Lee HY, Suh M, Park B, Song SH et al Effectiveness of the Korean National Cancer Screening Program in Reducing Gastric Cancer Mortality. Gastroenterology. 2017 5;152(6):1319–1328.e7. 10.1053/j.gastro.2017.01.029 28147224

[21] Kayamba V, Sinkala E, Mwanamakondo S, Soko R, Kawimbe B, Amadi B et al Trends in upper gastrointestinal diagnosis over four decades in Lusaka, Zambia: a retrospective analysis of endoscopic findings. *BMC Gastroenterol*. 2015; 15(1): 127. 26444265 10.1186/s12876-015-0353-8 PMC4596361

[22] Global Health Workforce alliance, http://www.who.int/workforcealliance/countries/zmb/en/, accessed on 19th December 2017.

[23] Colak S, Erdogan MO, Sekban H, Afacan MA, Uras AR, Ibrahim A et al Emergency diagnosis of upper gastrointestinal bleeding by detection of haemoglobin in nasogastric aspirate. J Int Med Res. 2013 12;41(6):1825–9. 10.1177/0300060513505516 24265333

[24] Liu H, Qiao P, Wu X, Wang L, Ao Y, Jia Z et al A smart capsule system of gastric occult blood detection. Biomed Mater Eng. 2014; 24(1):519–28. 10.3233/BME-130838 24211935

[25] Kelly P, Shawa T, Mwanamakondo S, Soko R, Smith G, Barclay GR et Gastric and intestinal barrier impairment in tropical enteropathy and HIV: limited impact of micronutrient supplementation during a randomised controlled trial. BMC Gastroenterol. 2010 7 6;10:72 10.1186/1471-230X-10-72 20604937 PMC2910659

