# Supplementary material for: Presence of blood in gastric juice: A sensitive marker for gastric cancer screening in a poor resource setting
Source: PLoS One. 2018 Oct 15;13(10):e0205185. doi: 10.1371/journal.pone.0205185 (PMC6188746; doi:10.1371/journal.pone.0205185)
Supplement: S1 Table — (DOCX) [file pone.0205185.s001.docx]

Supplementary Table

S1 Table

Testing the utility of urinary reagent strips for detection for blood in samples of with different pH levels

| pH | Colour change without blood | Colour change with blood |
| --- | --- | --- |
| 0 | Yes | Yes |
| 1 | Yes | Yes |
| 2 | Yes | Yes |
| 3 | No | Yes |
| 4 | No | Yes |
| 5 | No | Yes |
| 6 | No | Yes |
| 7 | No | Yes |
| 8 | No | Yes |
| 9 | No | Yes |
| 10 | No | Yes |
| 11 | No | Yes |
| 12 | Yes | Yes |
| 13 | Yes | Yes |
| 14 | Yes | Yes |

*Used in these experiments were hydrochloric acid (HCl) and sodium hydroxide (NaOH) to make solutions of various pH levels and these were tested with or with blood added
